# Ohio College of Dental Surgery—Its Commencement and Future Progress

**Published:** 1854-04

**Authors:** 


### Editorial.

---

OHIO COLLEGE OF DENTAL SURGERY—ITS COMMENCEMENT AND FUTURE PROSPECTS.

The commencement exercises of this Institution came off on Wednesday, 22d of February last, and the number of the Profession in attendance was unusually large, and a very good spirit prevailed. Altogether, it was an interesting occasion. Owing to the extreme stringency in the Money Market, the class was smaller than it would have been, yet the class numbered twenty-three. The graduating class was as follows:

George Watt, of Xenia, O.;

A. L. Brown, " "

B. N. Freeman, Seneca, O.;

Eli Collins, Connersville, Ia.;

J. B. Rottenstein, Galveston, Texas.;

M. W. S. Kendall, Cincinnati, O.;

E. A. Herman, Nashville, Tenn.

The honorary degree of D. D. S. was conferred on the following gentlemen:

P. G. C. Hunt, Indianapolis, Ia.;

M. DeCamp, Mansfield, O.;

J. W. Baxter, Warsaw, Ky.;

George Laurie, London, Eng.

The College Association had an interesting meeting, and we trust the result of their deliberations will be long remembered, and prove of lasting benefit to the Profession. By reference to the proceedings published in the present No. of the Register, it will be seen that they have authorized the erection of a new College edifice. The Committee appointed to superintend the building, have a plan of a stone front, which we think will be neat and appropriate, and we expect in a few days to close the contract for the building, which we are assured will be completed by the first of August. We shall have in the College, a General Lecture Room, an Anatomical and Chemical Lecture Room, a large and commodious Laboratory, two Infirmary Rooms, a Faculty Room; also, rooms for Library and Museum, and three Dissecting Rooms. The building throughout is to be finished in good style, with special reference to the wants of such an institution. We hope by our next issue to have a cut of the building, for the Register, and may then give a more detailed description of the building. so that the profession may know what we are doing for the cause of dental education.

The Mississippi Valley Association of Dental Surgeons, now hold their annual meetings just as the sessions of the College close, and we think the arrangement a happy one. It will bring more of the Profession together, and in this way may extend its usefulness. The last meeting was well attended, and more new members added than at any previous meeting. The discussions generally were of an interesting character, and general harmony prevailed.

It appeared to be a matter of general rejoicing that the American Society will hold their next meeting in this city. We fully expect the College to be in readiness.

---

### PROF. J. ALLEN'S RESIGNATION AND REMOVAL.

We learn that Dr. J. Allen, who has enjoyed the confidence and patronage of this community, as a dental practitioner, for more than twenty years, is about establishing a branch of his business in the city of New-York, at No. 30 Bond street, with a view of directing his exclusive attention to his improved style of work, still retaining a branch of his business in this city.

This change in his business operations, has rendered it necessary for him to resign his Professorship in the Ohio College of Dental Surgery, a chair which he has filled with *ability and general satisfaction; and we regret the necessity which impels* him to leave the school. Our wish is that he may meet with many friends and great success in New-York, where most of his time will be likely spent. We can commend him to our brethren in New-York, as a gentleman devoted to the Profession, courteous affable, and obliging.

---

The Chair of Operative Dentistry, in the Ohio College of Dental Surgery, made vacant by the resignation of Prof. J. Allen, we are happy to say has been already provided for. This shows the most admirable working of the Stock Association of the College, thus enabling such vacancies to be almost immedidiately filled. And in doing this, the wishes of many of our leading dentists have to be consulted; they making the nomination to the Board of Trustees, which indeed enables them to act with some advisement, in the filling of such vacancies. The nomination made for the Chair now vacated, was with great unanimity tendered Dr. J. Taft, of Xenia, Ohio; a gentleman who graduated in the school some six years since, and who has been engaged in practice for many years, having attained an enviable reputation as a writer and practitioner.

---

### CHARLES ABBY AND SON'S GOLD FOIL.

Our readers will observe the advertisement of these gentlemen, in the present No. of the Register. The reputation of their Foil is such that it needs no commendation from us. In speaking of Foil, the phrase is still familiar with the Profession, "As good as Abby's." We never hear of better.

### ARMSTRONG'S TEETH.

We have recently been shown some fine specimens of these gentlemen's teeth, of recent manufacture. The shading of the teeth is very good, and the color of the gum very life-like. There is one peculiarity in relation to the style of the gum teeth, we wish now to notice; which is, an adaptation to each other so as to—as much as possible—resemble block-work. The gum lengthens on the teeth from the central incisor back to the first bicuspid, and they have aimed to form them on the base, to require but little grinding. We see another good idea which they have adopted, which is the putting up of the four front upper teeth in sets, with the gum on the lateral incisors made to suit the fulness of the gum which is maintained by the cuspid tooth.

When they are enabled to supply stock for our market, we think they will find sale for many of their teeth.

### OUR ABSTRACTS.

We have been obliged in this No. to cut these down for want of room.

### THE SOCIETY'S PROCEEDINGS.

For sake of reference, we have published these, with the addresses, all in the present No. of the Register. This has left less room for other matter than we wished; and we would here remark, that Dr. Whiting's article on the use of nitrate of silver, was forwarded to us for presentation to the Society, but owing to the variety of matter then on hand, we forgot it until after the adjournment. The object of the Dr. was to draw out discussion upon the subject. The older members of the Mississippi Valley Association, remember with pleasure the Doctor's meeting with us years gone by, and hope next year to renew old acquaintance.

### DENTAL OFFICE FOR SALE.

We would draw attention to the "Card to Dentists." We have no doubt but that the situation is desirable, and the practice good. Any communication directed to our care, as noticed in the advertisement, will be sent immediately to the individual wishing to sell.

### STOCKHOLDERS OF THE OHIO COLLEGE OF DENTAL SURGERY.

For the information of those not in attendance at our last meeting, we would say, that the original stock has all been taken, and most all paid up. Those having paid the last installment, will in a short time receive by mail, or otherwise, certificates of stock. These certificates of stock, we would remark, were only designed to be temporary; the Association intending, after the new College is erected, to get an accurate cut of the same, and append to certificates of stock, gotten up in a very neat style, and then draw in the present ones. Of the twelve shares of additional stock authorized to be issued, five have already been taken; when the remaining seven are sold, there will be fifty in all, and we presume no more will be at any time required.

A Text-Book of Anatomy, *and Guide in Dissections, for the use of Students of Medicine and Dental Surgery; by* Washington R. Handy, M. D., *Professor of Anatomy and Physiology in the Baltimore College of Dental Surgery, etc.*

This is a work much needed, particularly by Dental Students. It has been arranged with special reference to the Anatomical studies of the dental student. The anatomy of the head, and the nerves, glands, etc., which are often implicated in dental disease, have been treated at full and in such a manner as to meet the wants of our students. The teeth are treated as of some consequence in the general organization; and we are glad to see such a work published, and heartily commend it to the Profession.

It is from the enterprising publishers, Lindsay & Blakiston, of Philadelphia, and is gotten up with their usual care and taste.

It contains some two hundred and sixty-four illustrations. It is for sale in this city by Dr. J. M. Brown. Agent for the Publishers.

ANNALS OF SCIENCE.

This a monthly periodical of thirty-two pages, large, 8 vo., published by Hamilton L. Smith, A. M., of Cleveland, Ohio. We are in the receipt of the first No. of Vol. 2. This is full of interesting matter—that which would interest the man of science. It should meet with an extensive circulation. It is really what it imports to be, the "Annals of Science." Subscription price, $1 00, paid in advance.

THE POPULAR EDUCATOR, AND ILLUSTRATED MAGAZINE OF ART.

We are in receipt of the last numbers of both these works. Some of the illustrations in the Magazine are rich, and, of themselves more than pay for the work. It is also full of interesting matter.

The Popular Educator contains articles on Language, Natural History, Mathematics, Fine Arts, Anthropology, Political Science, etc. These articles are well written and full of scientific matter.

